# Supplementary material for: Respiratory Distress Complicating Falciparum Malaria Imported to Berlin, Germany: Incidence, Burden, and Risk Factors
Source: Microorganisms. 2023 Jun 14;11(6):1579. doi: 10.3390/microorganisms11061579 (PMC10302277; doi:10.3390/microorganisms11061579)
Supplement: Supplementary file 1 [file microorganisms-11-01579-s001.zip › microorganisms-2401145-supplementary.pdf]

Supplementary Materials

Supplementary File S1: Statistical analysis

Required R packages:

```
> install.packages("survival")
> install.packages("survminer")
> install.packages("rstanarm")
> install.packages("bayestestR")
> install.packages("insight")
> install.packages("BayesFactor")
> install.packages("poorman")
> install.packages("modelbased")
> install.packages("performance")
> install.packages("logistf")
> install.packages("corrplot")
> install.packages("gplots")

> library(rstanarm)
> library(bayestestR)
> library(insight)
> library(BayesFactor)
> library(poorman)
> library(survival)
> library(survminer)
> library(tidyverse)
> library(caret)
> library(leaps)
> library(ggplot2)
> library(modelbased)
> library(gplots)
```

Web resources for the statistical analysis

- easystats.github.io (bayestestR)
- bayesrulesbook.com
- Bayesian Analysis in R, Author: Marissa Barlaz
- Bayesian Regression Models in R: Choosing informative priors in rstanarm

- STHDA.com (Statistical Tools for High Throughput Data Analysis)

Note: exp(0.74): 0.74 -> e^x = 2.09; Umkehrfunktion: ln -> 2.09 = 0.74

Guidelines for prior use:

- rstanarm attempts to make priors weakly informative by default by internally adjusting the scales of the priors. The phrase "weakly informative" is implicitly in comparison to a default flat (i.e., non-informative) prior.
- A weakly informative prior rules out unreasonable parameter values but is not so strong as to rule out values that might make sense.
- However, if the data are weak, a "weakly informative prior" will strongly influence the posterior inference. Thus, if there is not much prior information, a sensitivity analysis (where the prior distributions are changed) should be undertaken to make sure that prior choice is not unduly influencing inference.
- The idea of using weakly informative priors rather than fully informative ones is that the loss in precision by making the prior a bit too weak (compared to the true population distribution of parameters or the current expert state of knowledge) is less serious than the gain in robustness by including parts of parameter space that might be relevant.
- When informative priors are used, their choice should be explicitly; a sentence about each parameter in the model should be available.

Literature research: Important risk factors and modifiers for ARDS:.

Factor	Frequency (%) according to predisposing condition	Coefficient,	Odds ratio (CI)	Ref(s)	Included or excluded in the present analysis
Sepsis	6.8	0.37	1.44 (X-2.39)	Gajic, O, Am J Respir Crit Care Med, 2011	Included
Pneumonia	8.3	0.83	2.29 (X-3.82)	Gajic, O	Included
Shock	17.9	0.77	2.16 (X-3.74)	Gajic, O	Included
Aspiration	16.5	0.79	2.20 (X-4.26)<br>51 (7.1-369	Gajic, O<br>Ahmed AH, Crit Care Med, 2014	Included
Alcoholism			2.0-3.0		Included
History of smoking					Not included (due to probable reporting bias)
Hypoalbuminaemia (<35 g/L)					Included
Obesity					Included
Diabetes					Included
Age					Included
Pre-existing lung disease					Included
Chemotherapy					Included
(Massive) transfusion					
High FiO_2 >0.35 (>4 L/min.)					Not included (due to autocorrelation, part of the ARDS definition)
Tachypnea >30/min.					Not included (because autocorrelation, part of the ARDS definition
SpO_2 <95%					Not included (because of autocorrelation, part of the ARDS definition
Metabolic acidosis (pH <7.35)				Gajic, O.	Included

Additional predisposing factors in malaria (Maguire GP, J Infect Dis, 2005): SAPS II score, ARF, unarousable coma, metabolic acidosis, number of complications, bacterial co-infection, septic shock

1. Descriptive analysis:

**Parameter**	**Result**
Total number of cases treated in the institution during the study period	*n*=558 (representing 7.1% of the 7.866 cases notified in Germany during the study period)
Cases excluded	*n*=22 (all: individuals treated more than once in the institution; only the first malaria episode was included in the analysis). Figure S1 = flowchart
Proportion of patients with severe malaria according to the 2015 WHO criteria (Table S1)	*n*=55/536 (10.3%)
Number of cases admitted to the ICU	*n*=68/536 (12.7%; Figure S1 = flowchart):<br>*n*=41 (60.3%) with severe (SM) and<br>*n*=27 (39.7%) with uncomplicated disease (UM) according to the 2015 WHO criteria<br>*n*=14 cases with severe disease were treated on general wards because of sufficient clinical stability (n=11/14 of whom originated from a country of endemicity)
Reasons for ICU admission other than ≥1 criterion for severe malaria according to the 2015 WHO definition	During the study period definition of severe malaria changed three times (in 2006, 2010, and 2015). Therefore, prior to 2015 patients were admitted to the ICU not meeting the actual WHO definition.<br>Other cases required ICU admission due to advanced disease severity without strictly meeting the definition, e.g., non-immune individuals with high parasitaemias ≤10%.<br>In addition to the criteria of severe malaria other life-threatening conditions such as advanced hyponatraemia

	(Na^+ <115 mmol/L) or severe congestive heart failure also required ICU admission in individual cases.
LOSICU of the whole cohort	Median of 2.5 days (61 hours, IQR: 38-91 hours), total range: 9-644 hours, total of 6341 hours (264 patient days; Figure S2 = bar chart of LOSICU)
LOSICU of the 27 uncomplicated cases	Median of 38 hours, IQR: 21-59 hours, range: 9-111 hours
LOSICU of the 41 patients with severe malaria	Median of 66 hours, IQR: 52-138 hours, range: 14-644 hours, p<0.0001
Management	
Number of cases treated with artemisinin-based regimens	n=33/68 (48.5%)
Fluid management	Day 1: median of 2.1 (IQR 1.6-3.1, range 0.7-6.9) mL/kg/h<br>Day 2: median of 1.4 (IQR 0.9-2.3, range 0.4-6.1) mL/kg/h
Vasopressors needed	n=13/68 (19.1%)
Renal replacement therapy (RRT) needed	n=7/68 (10.3%)<br>Total days on dialysis: 51<br>Median time to RRT initiation: 20 hours
Co-infections (Table S2)	Total n=19/68 (28.0%) identified<br>Community-acquired co-infection: n=14/68 (20.6%)<br>Healthcare-associated co-infection: n=8/68 (11.8%)<br>Either type: n=2/68 (2.9%)<br>Two simultaneous community-acquired co-infections: n=1/68 (1.5%; P. vivax and HBV)

Median time to establishment of diagnosis of respiratory distress (Table. 1)	Median 17 hours, IQR 2-70 hours, range 0-144 hours<br>APO: 17 (IQR 2;34) hours<br>ARDS: 51 (IQR 15;89) hours
Low-flow oxygen therapy only	*n*=2 (2.9%)
Mechanical ventilation needed	*n*=9/68 (13.2%)<br>non-invasive mechanical ventilation (NIV) needed: *n*=4/68 (5.9%)<br>invasive mechanical ventilation (IV) needed: *n*=5/68 (7.4%)<br>Total hours on mechanical ventilation: 950 hours<br>Weaning from mechanical ventilation was uncomplicated in all cases
Number of malaria-specific complications on admission according to the WHO 2015 definition in all cases	Median 1, IQR 0-2 (range 0–9)
Number of total malaria-specific complications on admission in cases diagnosed with AOP/ARDS	Median 4, IQR 3–6 (range 1–9); Table S3
Number of fatal cases	*n*=0 (0%)

Table S1. Definition of individual malaria-specific complications and their frequency in the study population.

Criterion	Definition	Patients affected, *n* (%)
Jaundice	Plasma or serum bilirubin >3 mg/dL with parasitaemia >100.000/µL	21 (30.9)
Hyperparasitaemia	>10% parasitized erythrocytes	18 (26.5)
Decompensated shock	Systolic blood pressure <80 mmHg with need for norepinephrine dosages >0.05 µg/kg/min. to maintain mean arterial blood pressure >65 mmHg despite adequate hydration	12 (17.7)
Renal impairment	Plasma or serum creatinine >3 mg/dL or blood urea >120 mg/dL	12 (17.7)
Respiratory distress	Oxygen saturation on room air <92%, respiratory rate >30/min., and	11 (16.2)
Acute pulmonary oedema (APO)	Oxygen saturation on room air <92% and respiratory rate >30/min. together with $PaO_2/FiO_2 \leq 300$ mmHg and bilateral opacities on chest imaging	6 (8.8)
Acute respiratory distress syndrome (ARDS)	Lung injury within 1 week of admission with progression of respiratory symptoms; bilateral opacities on chest imaging not explained by other lung	5 (7.4)

	pathologies; respiratory failure not explained by heart failure or volume overload; PaO_2/FiO_2 ≤300 mmHg under a minimum PEEP of 5 cmH_2O (applied by non-invasive or invasive ventilation)	
Significant bleeding	Including recurrent or prolonged bleeding from the nose, gums, venepuncture sites, haematemesis, or malaena	10 (14.7)
Severe malarial anaemia	Haemoglobin level <7 g/dL and/or haematocrit <20% with parasitaemia >0.5%	10 (14.7)
Coma	Glasgow coma scale (GCS) <11	7 (10.3)
Metabolic acidosis	Base deficit >8 mmol/L and/or bicarbonate <15 mmol/L and/or venous plasma lactate ≥5 mmol/L or ≥45 mg/dL	7 (10.3)
Hypoglycaemia	Blood glucose level <40 mg/dL	1 (1.5)
Convulsions	>2 convulsions within 24 hours	0 (0.0)

Abbreviations: PaO_2/FiO_2, oxygenation index; PEEP, positive end expiratory pressure.

Table S2. Co-infections identified in the study population during ICU stay:.

Type of co-infection	Isolated species	Frequency
Community-acquired co-infections (*n*=13)		
Chronic viral co-infection	HIV<br>Hepatitis C	7<br>1
Mixed malaria	*P. malariae* + *P. falciparum*	2
Other travel-associated co-infection	*L. interrogans*<br>Dengue virus	1<br>1
≥1 Co-infection	Hepatitis B +<br>*P. vivax* + *P. falciparum*	1
Healthcare-associated co-infections (n=8)		
Central-line-associated blood-stream infection (CLABSI)	*S. epidermidis*	3
Aspiration/pneumonia	*S. aureus (MSSA)*<br>*S. aureus (MSSA)* and *P. aeruginosa*	1<br>1
Catheter-associated urinary tract infection (CAUTI)	*E. coli*	3

Abbreviations: HBV, Hepatitis B; HCV, Hepatitis C; HIV, Human immunodeficiency virus; MSSA, methicillin-sensitive staphylococcus aureus

2. Explorative analysis

2.1. Association of individual malaria-specific complications with length of stay on ICU (LOSICU = Response/dependent variable)

2.1.1. Univariate Association of individual malaria-specific complications with LOSICU

Import dataset "MicroRDFILE", Sheet "ICU patients (68)"

R commands:

```
> install.packages("survival")

> install.packages("survminer")


> library("survival")

> library("survminer")


> head(MicroRDFILE)
```

Example:

```
> res.cox <- coxph(Surv(LOSICU, LOSICUMED) ~ JaundiceWHOo, data = MicroRDFILE)
> res.cox
> summary(res.cox)

Call:
coxph(formula = Surv(LOSICU, LOSICUMED) ~ JaundiceWHOo, data = MicroRDFILE)

                coef exp(coef) se(coef)      z     p
JaundiceWHOo -0.3917    0.6759   0.3652 -1.073 0.283

Likelihood ratio test=1.18  on 1 df, p=0.2765
n= 68, number of events= 34
> summary(res.cox)
Call:
coxph(formula = Surv(LOSICU, LOSICUMED) ~ JaundiceWHOo, data = MicroRDFILE)

  n= 68, number of events= 34

                coef exp(coef) se(coef)      z Pr(>|z|)
JaundiceWHOo -0.3917    0.6759   0.3652 -1.073    0.283

             exp(coef) exp(-coef) lower .95 upper .95
JaundiceWHOo    0.6759       1.48    0.3304     1.383

Concordance= 0.552  (se = 0.051 )
Likelihood ratio test= 1.18  on 1 df,   p=0.3
Wald test            = 1.15  on 1 df,   p=0.3
Score (logrank) test = 1.16  on 1 df,   p=0.3
```

Table S3. Association of individual malaria-specific complications with length of ICU stay in 68 patients requiring intensive care.

Criterion	Patients affected, *n* (%)	Number of total malaria-specific complications on admission, median (IQR)	Median LOS-ICU (IQR)	Sensitivity analysis: Hazard Ratio (95%CI) for ICU discharge by 38 hours	P value	Sensitivity analysis: Hazard Ratio (95%CI) for ICU discharge by 61 hours	P value	Hazard Ratio (95%CI) for ICU discharge by 91 hours	P value
Jaundice	21 (30.9)	2 (1;5)	64 (48;122)	0.78 (0.44-1.39)	0.405	0.68 (0.33-1.38)	0.283	1.00 (0.37-2.67)	0.993
Hyperparasitaemia	18 (26.5)	2 (1;4)	66 (40;109)	0.84 (0.45-1.57)	0.586	0.77 (0.36-1.67)	0.515	0.98 (0.34-2.85)	0.968
Decompensated shock	12 (17.7)	4 (4;6)	160(82;332)	0.28 (0.13-0.60)	0.001	0.31 (0.13-0.73)	0.008	0.29 (0.09-0.98)	0.045
Renal impairment	12 (17.7)	5 (3;6)	188 (109;258)	0.288 (0.14-0.58)	<0.001	0.38 (0.18-0.80)	0.011	0.60 (0.22-1.64)	0.319

Respiratory distress	11 (16.2)	4 (3;6)	200 (146;390)	0.17 (0.07-0.42)	<0.001	0.17 (0.06-0.44)	<0.001	0.21 (0.06-0.67)	0.009
APO	6 (8.8)	5 (3;7)	146 (77;192)	0.47 (0.19-1.21)	0.118	0.48 (0.17-1.38)	0.175	0.63 (0.18-2.23)	0.47
ARDS	5 (7.4)	4 (3;4)	275 (238; 504)	0.24 (0.09-0.65)	0.005	0.28 (0.10-0.77)	0.013	0.41 (0.14-1.23)	0.111
Significant bleeding	10 (14.7)	4 (2;6)	115 (67;134)	0.52 (0.25-1.10)	0.084	0.63 (0.28-1.41)	0.262	0.91 (0.33-2.55)	0.858
Severe malarial anaemia	10 (14.7)	4 (2;6)	139 (64;428)	0.30 (0.12-0.73)	0.008	0.32 (0.12-0.85)	0.023	0.17 (0.04-0.77)	0.021
Coma	7 (10.3)	6 (4;7)	195(109;390)	0.36 (0.16-0.82)	0.016	0.45 (0.19-1.08)	0.073	0.70 (0.25-2.00)	0.51
Metabolic acidosis	7 (10.3)	6 (4;7)	195 (124; 352)	0.32 (0.13-0.77)	0.011	0.40 (0.16-1.00)	0.050	0.58 (0.20-1.69)	0.317
Hypoglycaemia	1 (1.5)	9 (9;9)	195 (195;195)	0.47 (0.19-1.21)	0.118	0.48 (0.17-1.38)	0.175	1.20 (0.15-9.56)	0.862
Convulsions	0 (0.0)	-	-	-	-	-	-	-	-

Abbreviations: APO, acute pulmonary oedema; ARDS, acute respiratory distress syndrome.

⇒ ***Interpretation:*** *respiratory distress was the complication with the strongest association with ICU-LOS in univariate analysis in the cohort.*

2.1.2. Variable selection for LOSICU employing the best subset selection method:

Import dataset "MicroRDFILE", Sheet "LOSICU BESS"

R commands:

```
> library(tidyverse)

> library(caret)

> library(leaps)

> models <- regsubsets(LOSICU~., data = MicroRDFILE, nvmax = 10)

> summary(models)
```

```
Subset selection object
Call: regsubsets.formula(LOSICU ~ ., data = MicroRDFILE, nvmax = 10)
10 Variables  (and intercept)
                   Forced in Forced out
JaundiceWHOo           FALSE      FALSE
Hyperparasitemia10     FALSE      FALSE
ShockWHOo              FALSE      FALSE
RenalimpWHOo           FALSE      FALSE
RespdistressWHOo       FALSE      FALSE
BleedingWHOo           FALSE      FALSE
Anaemiao               FALSE      FALSE
ComaWHOo               FALSE      FALSE
AcidosisWHOo           FALSE      FALSE
HypoglyWHOo            FALSE      FALSE
1 subsets of each size up to 10
Selection Algorithm: exhaustive
          JaundiceWHOo Hyperparasitemia10 ShockWHOo RenalimpWHOo RespdistressWHOo BleedingWHOo Anaemiao ComaWHOo AcidosisWHOo HypoglyWHOo
1  ( 1 )  " "          " "                " "       " "          "*"              " "          " "      " "      " "          " "
2  ( 1 )  " "          " "                " "       " "          "*"              " "          "*"      " "      " "          " "
```

```
3  ( 1 )  " "        " "            "*"     " "       "*"          " "     "*"    " "    " "       " "
4  ( 1 )  " "        " "            " "     " "       "*"          "*"     "*"    " "    " "       "*"
5  ( 1 )  " "        " "            "*"     " "       "*"          "*"     "*"    " "    " "       "*"
6  ( 1 )  " "        " "            "*"     "*"       "*"          "*"     "*"    " "    " "       "*"
7  ( 1 )  "*"        " "            "*"     "*"       "*"          "*"     "*"    " "    " "       "*"
8  ( 1 )  "*"        "*"            "*"     "*"       "*"          "*"     "*"    " "    " "       "*"
9  ( 1 )  "*"        "*"            "*"     "*"       "*"          "*"     "*"    " "    "*"       "*"
10  ( 1 ) "*"        "*"            "*"     "*"       "*"          "*"     "*"    "*"    "*"       "*"
```

⇒ ***Interpretation:*** *Employing the best subset selection method, respiratory distress was the complication with the strongest association with LOSICU in the cohort, too.*

2.1.3. Multivariate cox hazard proportional regression (adjusted for age, provenience, and artemisinin therapy)

Import dataset "MicroRDFILE", Sheet "ICU patients (68)"

R commands:

```
> library(survival)

> library(survminer)

res.cox <- coxph(Surv(LOSICU, LOSICUMED) ~ Age + Endemic + Artemisinins + RespdistressWHOo +
AnemiaWHOo + ShockWHOo, data = MicroRDFILE)

> summary(res.cox)

Call:

coxph(formula = Surv(LOSICU, LOSICUMED) ~ Age + Endemic + Artemisinins +

    RespdistressWHOo + AnemiaWHOo + ShockWHOo, data = APORFILE_02_23)


  n= 68, number of events= 34


                     coef exp(coef) se(coef)      z Pr(>|z|)

Age              -0.01076   0.98930  0.01858 -0.579   0.5626

Endemic          -0.08723   0.91647  0.55801 -0.156   0.8758

Artemisinins      0.86206   2.36804  0.43348  1.989   0.0467 *

RespdistressWHOo -1.42737   0.23994  0.58244 -2.451   0.0143 *

AnemiaWHOo       -0.16891   0.84458  0.65052 -0.260   0.7951

ShockWHOo        -0.73719   0.47845  0.48555 -1.518   0.1290

---

Signif. codes:  0 '***' 0.001 '**' 0.01 '*' 0.05 '.' 0.1 ' ' 1


                 exp(coef) exp(-coef) lower .95 upper .95

Age                 0.9893     1.0108   0.95391    1.0260

Endemic             0.9165     1.0911   0.30700    2.7359

Artemisinins        2.3680     0.4223   1.01254    5.5381
```

```
RespdistressWHOo    0.2399     4.1677   0.07662    0.7514
AnemiaWHOo          0.8446     1.1840   0.23601    3.0225
ShockWHOo           0.4785     2.0901   0.18473    1.2392

Concordance= 0.754  (se = 0.056 )
Likelihood ratio test= 21.88  on 6 df,   p=0.001
Wald test            = 16.74  on 6 df,   p=0.01
Score (logrank) test = 19.63  on 6 df,   p=0.003
```

⇒ ***Interpretation:*** *Employing multivariate cox proportional hazard regression, respiratory distress was the only complication significantly associated with LOSICU after adjusting for age, provenience, and treatment with artemisinin regimen as important potential confounders.*

Model diagnostics - proportional hazard assumption:

```
> test.ph <- cox.zph(res.cox)
> test.ph
                  chisq df      p
Age               1.364  1 0.2428
Endemic           7.936  1 0.0048
Artemisinins      0.665  1 0.4150
RespdistressWHOo 1.077  1 0.2993
AnemiaWHOo        0.142  1 0.7061
ShockWHOo         1.134  1 0.2870
GLOBAL            9.776  6 0.1344
```

⇒ ***Interpretation:*** *The test is not statistically significant for the covariates (except from "Endemic") and the global test. Therefore, the proportional hazards can be assumed.*

Model diagnostics - Schoenfield residuals:

```
>ggcoxzph(test.ph)
```

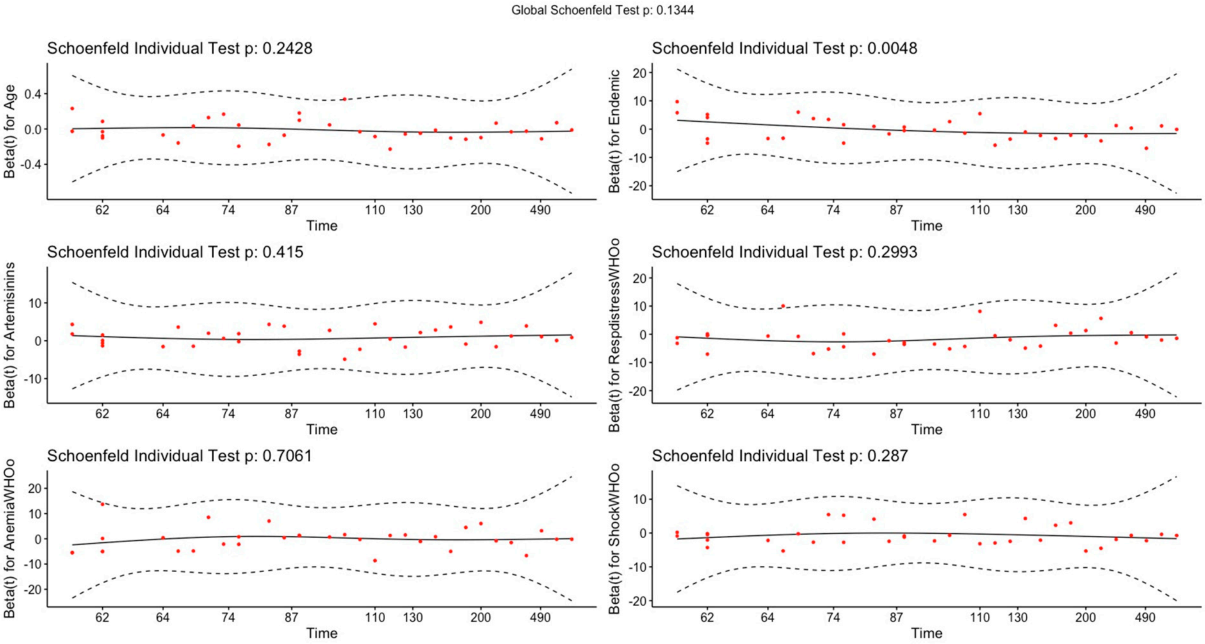

Figure S1. Schoenfiled residuals.

Model diagnostics – influential observations (dfbeta values):

```
> ggcoxdiagnostics(res.cox, type = "dfbeta", linear.predictions = FALSE,
ggtheme = theme_bw())
```

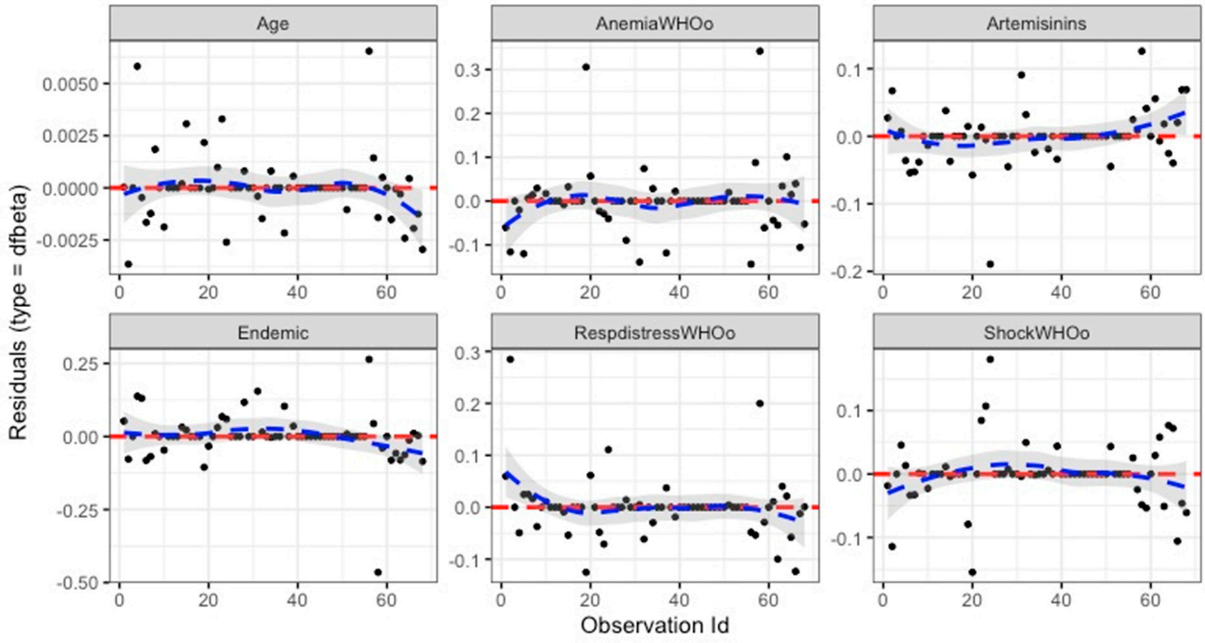

Figure S2. Influential observations.

2.2 Risk factors for Respiratory distress

Response/dependent variable = RespdistressWHOo

```
> install.packages("rstanarm")
> install.packages("bayestestR")
> install.packages("insight")
> install.packages("BayesFactor")
> install.packages("modelbased")
> install.packages("performance")
> install.packages("logistf")
> install.packages("patchwork")
> install.packages("poorman")

> library(rstanarm)
> library(bayestestR)
> library(insight)
> library(BayesFactor)
> library(ggplot2)
> library(modelbased)
> library(performance)
> library(logistf)
> library(patchwork)
> library(poorman)

> View(MicroRDFILE)
> head(MicroRDFILE)
```

Example for a standard logistic regression:

```
> model0 <- glm(RespdistressWHOo ~ ShockWHOo, data = MicroRDFILE, family = binomial)
> summary(model0)
Call:
glm(formula = RespdistressWHOo ~ ShockWHOo, data = MicroRDFILE,
    family = binomial)

Residuals:
     Min        1Q    Median        3Q       Max
-0.58333 -0.07143 -0.07143 -0.07143   0.92857

Coefficients:
```

```
            Estimate Std. Error t value Pr(>|t|)
(Intercept)  0.07143    0.04236   1.686   0.0964 .
ShockWHOo    0.51190    0.10083   5.077 3.36e-06 ***
---
Signif. codes:  0 '***' 0.001 '**' 0.01 '*' 0.05 '.' 0.1 ' ' 1

Residual standard error: 0.317 on 66 degrees of freedom
Multiple R-squared:  0.2809,  Adjusted R-squared:   0.27
F-statistic: 25.78 on 1 and 66 DF,  p-value: 3.359e-06

> exp(cbind(OR=coef(model0),confint(model0)))
                    OR      2.5 %     97.5 %
(Intercept)  0.07692308 0.02326494  0.1879144
ShockWHOo   18.20000000 4.17204737 94.3103640
```

Example for a penalized (Firth) logistic regression:

```
> library(logistf)
> model1<-logistf(data=MicroRDFILE, RespdistressWHOo ~ ShockWHOo, firth=TRUE, pl=TRUE)
> summary(model1)
> exp(cbind(OR=coef(model1), confint(model1)))

logistf(formula = RespdistressWHOo ~ ShockWHOo, data = MicroRDFILE,
    pl = TRUE, firth = TRUE)

Model fitted by Penalized ML
Coefficients:
                 coef  se(coef) lower 0.95 upper 0.95    Chisq            p method
(Intercept) -2.456736 0.4911923  -3.575405  -1.603701 47.53300 5.408562e-12      2
ShockWHOo    2.766891 0.7459366   1.357095   4.325223 14.94151 1.108959e-04      2

Method: 1-Wald, 2-Profile penalized log-likelihood, 3-None

Likelihood ratio test=14.94151 on 1 df, p=0.0001108959, n=68
Wald test = 25.32101 on 1 df, p = 4.853936e-07

> exp(cbind(OR=coef(model1), confint(model1)))
                       OR  Lower 95%  Upper 95%
(Intercept)  0.08571429 0.02800409  0.2011506
ShockWHOo   15.90909091 3.88488955 75.5823749
```

Example for a Bayesian logistic regression using weakly informative (default) priors (including prior description)

```
> model2 <- stan_glm(RespdistressWHOo ~ ShockWHOo, data = MicroRDFILE, family = binomial)
> posteriors <- describe_posterior(model2)
> print_md(posteriors, digits = 2)
> ps1 <- prior_summary(model2)
> ps1$prior
> check_model(model2)
```

```
SAMPLING FOR MODEL 'bernoulli' NOW (CHAIN 1).
Chain 1:
Chain 1: Gradient evaluation took 0.000102 seconds
Chain 1: 1000 transitions using 10 leapfrog steps per transition would take 1.02 seconds.
Chain 1: Adjust your expectations accordingly!
Chain 1:
Chain 1:
Chain 1: Iteration:    1 / 2000 [  0%]  (Warmup)
Chain 1: Iteration:  200 / 2000 [ 10%]  (Warmup)
Chain 1: Iteration:  400 / 2000 [ 20%]  (Warmup)
Chain 1: Iteration:  600 / 2000 [ 30%]  (Warmup)
Chain 1: Iteration:  800 / 2000 [ 40%]  (Warmup)
Chain 1: Iteration: 1000 / 2000 [ 50%]  (Warmup)
Chain 1: Iteration: 1001 / 2000 [ 50%]  (Sampling)
Chain 1: Iteration: 1200 / 2000 [ 60%]  (Sampling)
Chain 1: Iteration: 1400 / 2000 [ 70%]  (Sampling)
Chain 1: Iteration: 1600 / 2000 [ 80%]  (Sampling)
Chain 1: Iteration: 1800 / 2000 [ 90%]  (Sampling)
Chain 1: Iteration: 2000 / 2000 [100%]  (Sampling)
Chain 1:
Chain 1:  Elapsed Time: 0.042424 seconds (Warm-up)
Chain 1:                0.043371 seconds (Sampling)
Chain 1:                0.085795 seconds (Total)
Chain 1:

SAMPLING FOR MODEL 'bernoulli' NOW (CHAIN 2).
Chain 2:
Chain 2: Gradient evaluation took 3.2e-05 seconds
```

```
Chain 2: 1000 transitions using 10 leapfrog steps per transition would take 0.32 seconds.
Chain 2: Adjust your expectations accordingly!
Chain 2:
Chain 2:
Chain 2: Iteration:    1 / 2000 [  0%]  (Warmup)
Chain 2: Iteration:  200 / 2000 [ 10%]  (Warmup)
Chain 2: Iteration:  400 / 2000 [ 20%]  (Warmup)
Chain 2: Iteration:  600 / 2000 [ 30%]  (Warmup)
Chain 2: Iteration:  800 / 2000 [ 40%]  (Warmup)
Chain 2: Iteration: 1000 / 2000 [ 50%]  (Warmup)
Chain 2: Iteration: 1001 / 2000 [ 50%]  (Sampling)
Chain 2: Iteration: 1200 / 2000 [ 60%]  (Sampling)
Chain 2: Iteration: 1400 / 2000 [ 70%]  (Sampling)
Chain 2: Iteration: 1600 / 2000 [ 80%]  (Sampling)
Chain 2: Iteration: 1800 / 2000 [ 90%]  (Sampling)
Chain 2: Iteration: 2000 / 2000 [100%]  (Sampling)
Chain 2:
Chain 2:  Elapsed Time: 0.048317 seconds (Warm-up)
Chain 2:                0.053514 seconds (Sampling)
Chain 2:                0.101831 seconds (Total)
Chain 2:


SAMPLING FOR MODEL 'bernoulli' NOW (CHAIN 3).
Chain 3:
Chain 3: Gradient evaluation took 3.5e-05 seconds
Chain 3: 1000 transitions using 10 leapfrog steps per transition would take 0.35 seconds.
Chain 3: Adjust your expectations accordingly!
Chain 3:
Chain 3:
Chain 3: Iteration:    1 / 2000 [  0%]  (Warmup)
Chain 3: Iteration:  200 / 2000 [ 10%]  (Warmup)
Chain 3: Iteration:  400 / 2000 [ 20%]  (Warmup)
Chain 3: Iteration:  600 / 2000 [ 30%]  (Warmup)
Chain 3: Iteration:  800 / 2000 [ 40%]  (Warmup)
Chain 3: Iteration: 1000 / 2000 [ 50%]  (Warmup)
Chain 3: Iteration: 1001 / 2000 [ 50%]  (Sampling)
Chain 3: Iteration: 1200 / 2000 [ 60%]  (Sampling)
Chain 3: Iteration: 1400 / 2000 [ 70%]  (Sampling)
Chain 3: Iteration: 1600 / 2000 [ 80%]  (Sampling)
```

```
Chain 3: Iteration: 1800 / 2000 [ 90%]  (Sampling)
Chain 3: Iteration: 2000 / 2000 [100%]  (Sampling)
Chain 3:
Chain 3:  Elapsed Time: 0.042735 seconds (Warm-up)
Chain 3:                0.052772 seconds (Sampling)
Chain 3:                0.095507 seconds (Total)
Chain 3:


SAMPLING FOR MODEL 'bernoulli' NOW (CHAIN 4).
Chain 4:
Chain 4: Gradient evaluation took 2e-05 seconds
Chain 4: 1000 transitions using 10 leapfrog steps per transition would take 0.2 seconds.
Chain 4: Adjust your expectations accordingly!
Chain 4:
Chain 4:
Chain 4: Iteration:    1 / 2000 [  0%]  (Warmup)
Chain 4: Iteration:  200 / 2000 [ 10%]  (Warmup)
Chain 4: Iteration:  400 / 2000 [ 20%]  (Warmup)
Chain 4: Iteration:  600 / 2000 [ 30%]  (Warmup)
Chain 4: Iteration:  800 / 2000 [ 40%]  (Warmup)
Chain 4: Iteration: 1000 / 2000 [ 50%]  (Warmup)
Chain 4: Iteration: 1001 / 2000 [ 50%]  (Sampling)
Chain 4: Iteration: 1200 / 2000 [ 60%]  (Sampling)
Chain 4: Iteration: 1400 / 2000 [ 70%]  (Sampling)
Chain 4: Iteration: 1600 / 2000 [ 80%]  (Sampling)
Chain 4: Iteration: 1800 / 2000 [ 90%]  (Sampling)
Chain 4: Iteration: 2000 / 2000 [100%]  (Sampling)
Chain 4:
Chain 4:  Elapsed Time: 0.043438 seconds (Warm-up)
Chain 4:                0.051199 seconds (Sampling)
Chain 4:                0.094637 seconds (Total)
Chain 4:
> posteriors <- describe_posterior(model2)
> print_md(posteriors, digits = 2)



Table: Summary of Posterior Distribution


|Parameter    | Median|        95% CI |   pd |          ROPE | % in ROPE| Rhat |     ESS |
```

```
|:-----------|------:|:--------------|:----|:-------------|---------:|:-----|:-------|
|(Intercept) |  -2.54|[-3.68, -1.68] |100% |[-0.10, 0.10] |        0%|1.002 |1581.00 |
|ShockWHOo    |   **2.89**|[ 1.47,  4.58] |100% |[-0.10, 0.10] |        0%|1.003 |1834.00 |

> ps1 <- prior_summary(model2)
> ps1$prior

$dist
[1] "normal"

$location
[1] 0

$scale
[1] 2.5

$adjusted_scale
[1] 6.509494

$df
NULL
```

Example for a Bayesian logistic regression using an informative prior without assumption on scale parameters:

```
> p_ShockWHOo_mid <- log(2)
> p_ShockWHOo_hi <- log(2.2)
> model3 <- stan_glm(RespdistressWHOo ~ ShockWHOo, data = MicroRDFILE, family = binomial("logit"))
  prior = normal(location = c(0, p_ShockWHOo_mid, p_ShockWHOo_hi, 0, 0, 0), scale = NULL)
> posteriors <- describe_posterior(model3)
> print_md(posteriors, digits = 2)
> ps1 <- prior_summary(model3)
> ps1$prior
> check_model(model3)

SAMPLING FOR MODEL 'bernoulli' NOW (CHAIN 1).
Chain 1:
Chain 1: Gradient evaluation took 1.6e-05 seconds
Chain 1: 1000 transitions using 10 leapfrog steps per transition would take 0.16 seconds.
```

```
Chain 1: Adjust your expectations accordingly!
Chain 1:
Chain 1:
Chain 1: Iteration:    1 / 2000 [  0%]  (Warmup)
Chain 1: Iteration:  200 / 2000 [ 10%]  (Warmup)
Chain 1: Iteration:  400 / 2000 [ 20%]  (Warmup)
Chain 1: Iteration:  600 / 2000 [ 30%]  (Warmup)
Chain 1: Iteration:  800 / 2000 [ 40%]  (Warmup)
Chain 1: Iteration: 1000 / 2000 [ 50%]  (Warmup)
Chain 1: Iteration: 1001 / 2000 [ 50%]  (Sampling)
Chain 1: Iteration: 1200 / 2000 [ 60%]  (Sampling)
Chain 1: Iteration: 1400 / 2000 [ 70%]  (Sampling)
Chain 1: Iteration: 1600 / 2000 [ 80%]  (Sampling)
Chain 1: Iteration: 1800 / 2000 [ 90%]  (Sampling)
Chain 1: Iteration: 2000 / 2000 [100%]  (Sampling)
Chain 1:
Chain 1:  Elapsed Time: 0.043549 seconds (Warm-up)
Chain 1:                0.043439 seconds (Sampling)
Chain 1:                0.086988 seconds (Total)
Chain 1:


SAMPLING FOR MODEL 'bernoulli' NOW (CHAIN 2).
Chain 2:
Chain 2: Gradient evaluation took 1.3e-05 seconds
Chain 2: 1000 transitions using 10 leapfrog steps per transition would take 0.13 seconds.
Chain 2: Adjust your expectations accordingly!
Chain 2:
Chain 2:
Chain 2: Iteration:    1 / 2000 [  0%]  (Warmup)
Chain 2: Iteration:  200 / 2000 [ 10%]  (Warmup)
Chain 2: Iteration:  400 / 2000 [ 20%]  (Warmup)
Chain 2: Iteration:  600 / 2000 [ 30%]  (Warmup)
Chain 2: Iteration:  800 / 2000 [ 40%]  (Warmup)
Chain 2: Iteration: 1000 / 2000 [ 50%]  (Warmup)
Chain 2: Iteration: 1001 / 2000 [ 50%]  (Sampling)
Chain 2: Iteration: 1200 / 2000 [ 60%]  (Sampling)
Chain 2: Iteration: 1400 / 2000 [ 70%]  (Sampling)
Chain 2: Iteration: 1600 / 2000 [ 80%]  (Sampling)
Chain 2: Iteration: 1800 / 2000 [ 90%]  (Sampling)
```

```
Chain 2: Iteration: 2000 / 2000 [100%]  (Sampling)
Chain 2:
Chain 2:  Elapsed Time: 0.041537 seconds (Warm-up)
Chain 2:                0.046005 seconds (Sampling)
Chain 2:                0.087542 seconds (Total)
Chain 2:

SAMPLING FOR MODEL 'bernoulli' NOW (CHAIN 3).
Chain 3:
Chain 3: Gradient evaluation took 1.7e-05 seconds
Chain 3: 1000 transitions using 10 leapfrog steps per transition would take 0.17 seconds.
Chain 3: Adjust your expectations accordingly!
Chain 3:
Chain 3:
Chain 3: Iteration:    1 / 2000 [  0%]  (Warmup)
Chain 3: Iteration:  200 / 2000 [ 10%]  (Warmup)
Chain 3: Iteration:  400 / 2000 [ 20%]  (Warmup)
Chain 3: Iteration:  600 / 2000 [ 30%]  (Warmup)
Chain 3: Iteration:  800 / 2000 [ 40%]  (Warmup)
Chain 3: Iteration: 1000 / 2000 [ 50%]  (Warmup)
Chain 3: Iteration: 1001 / 2000 [ 50%]  (Sampling)
Chain 3: Iteration: 1200 / 2000 [ 60%]  (Sampling)
Chain 3: Iteration: 1400 / 2000 [ 70%]  (Sampling)
Chain 3: Iteration: 1600 / 2000 [ 80%]  (Sampling)
Chain 3: Iteration: 1800 / 2000 [ 90%]  (Sampling)
Chain 3: Iteration: 2000 / 2000 [100%]  (Sampling)
Chain 3:
Chain 3:  Elapsed Time: 0.042986 seconds (Warm-up)
Chain 3:                0.053158 seconds (Sampling)
Chain 3:                0.096144 seconds (Total)
Chain 3:

SAMPLING FOR MODEL 'bernoulli' NOW (CHAIN 4).
Chain 4:
Chain 4: Gradient evaluation took 1.5e-05 seconds
Chain 4: 1000 transitions using 10 leapfrog steps per transition would take 0.15 seconds.
Chain 4: Adjust your expectations accordingly!
Chain 4:
Chain 4:
```

```
Chain 4: Iteration:    1 / 2000 [  0%]  (Warmup)
Chain 4: Iteration:  200 / 2000 [ 10%]  (Warmup)
Chain 4: Iteration:  400 / 2000 [ 20%]  (Warmup)
Chain 4: Iteration:  600 / 2000 [ 30%]  (Warmup)
Chain 4: Iteration:  800 / 2000 [ 40%]  (Warmup)
Chain 4: Iteration: 1000 / 2000 [ 50%]  (Warmup)
Chain 4: Iteration: 1001 / 2000 [ 50%]  (Sampling)
Chain 4: Iteration: 1200 / 2000 [ 60%]  (Sampling)
Chain 4: Iteration: 1400 / 2000 [ 70%]  (Sampling)
Chain 4: Iteration: 1600 / 2000 [ 80%]  (Sampling)
Chain 4: Iteration: 1800 / 2000 [ 90%]  (Sampling)
Chain 4: Iteration: 2000 / 2000 [100%]  (Sampling)
Chain 4:
Chain 4:  Elapsed Time: 0.045825 seconds (Warm-up)
Chain 4:                0.048221 seconds (Sampling)
Chain 4:                0.094046 seconds (Total)
Chain 4:
> prior = normal(location = c(0, p_ShockWHOo_mid, p_ShockWHOo_hi, 0, 0, 0), scale = NULL)
> posteriors <- describe_posterior(model3)
> print_md(posteriors, digits = 2)


Table: Summary of Posterior Distribution

|Parameter   | Median|        95% CI |  pd |         ROPE | % in ROPE| Rhat |    ESS |
|:-----------|------:|:--------------|:----|:-------------|---------:|:-----|:-------|
|(Intercept) |  -2.53|[-3.72, -1.68] |100% |[-0.10, 0.10] |        0%|1.000 |1773.00 |
|ShockWHOo   |   2.92|[ 1.48,  4.50] |100% |[-0.10, 0.10] |        0%|1.000 |2059.00 |
> ps1 <- prior_summary(model3)
> ps1$prior
$dist
[1] "normal"

$location
[1] 0

$scale
[1] 2.5
```

```
$adjusted_scale
[1] 6.509494

$df
NULL

> check_model(model3)
```

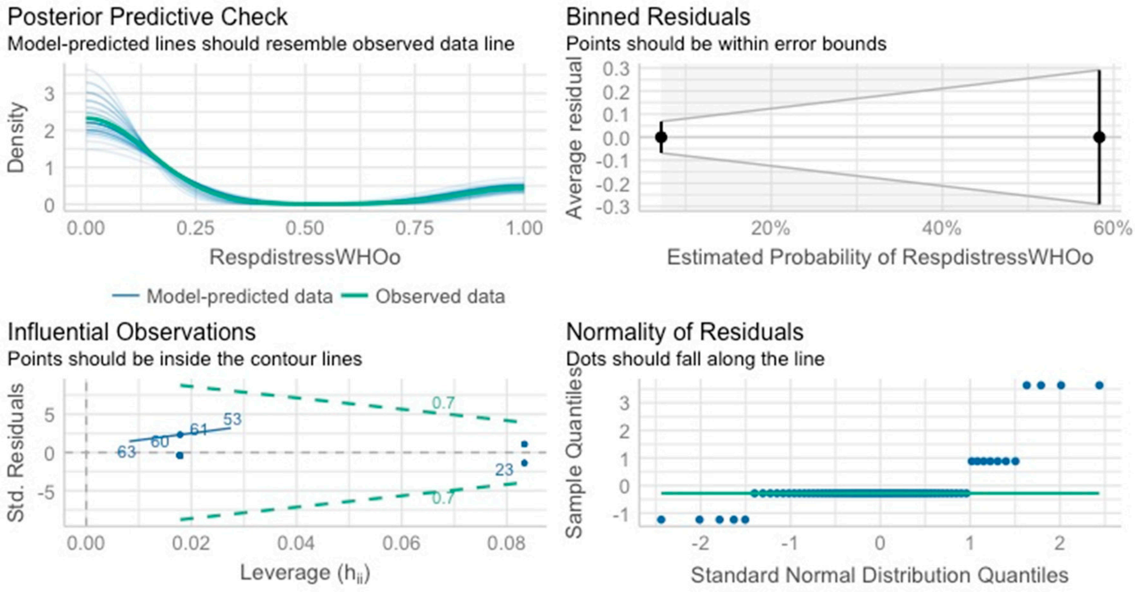

Figure S3. Example for model diagnostics of a Bayesian logistic regression.

Visualization of the prior distribution

```
> model <- stan_glm(RespdistressWHOo ~ ShockWHOo, data = MicroRDFILE, chains = 2, iter = 1000,
warmup = 250)
> posteriors <- get_parameters(model)
> head(posteriors)
> ggplot(posteriors, aes(x = ShockWHOo)) + geom_density(fill = "orange") + geom_vline(xintercept
= median(posteriors$ShockWHOo), color = "red", size = 1)
```

2.2.1 Univariate logistic regression for factors associated with development of respiratory distress:

Refer to Table 6 in the manuscript.

2.2.2. Best subset selection for selecting covariates for the final multivariable model:

R commands:

```
> library(tidyverse)
> library(caret)
> library(leaps)
```

```
> models <- regsubsets(RespdistressWHOo~., data = MicroRDFILE, nvmax = 14)
> summary(models)
```

```
Subset selection object
Call: regsubsets.formula(RespdistressWHOo ~ ., data = MicroRDFILE,
    nvmax = 14)
14 Variables  (and intercept)
              Forced in Forced out
RRTo              FALSE      FALSE
ComaWHOo          FALSE      FALSE
Coinf             FALSE      FALSE
ShockWHOo         FALSE      FALSE
RenalimpWHOo      FALSE      FALSE
Transfusion       FALSE      FALSE
AnemiaWHOo        FALSE      FALSE
Hypoalbo          FALSE      FALSE
FluidsIn1         FALSE      FALSE
FluidsIn2         FALSE      FALSE
CACCI             FALSE      FALSE
MinBEo            FALSE      FALSE
Bua               FALSE      FALSE
SAPSIIa           FALSE      FALSE
1 subsets of each size up to 14
Selection Algorithm: exhaustive
          RRTo ComaWHOo Coinf ShockWHOo RenalimpWHOo Transfusion AnemiaWHOo Hypoalbo FluidsIn1 FluidsIn2 CACCI MinBEo Bua SAPSIIa
1  ( 1 )  " "  " "      " "   " "       "*"          " "         " "        " "      " "       " "       " "   " "    " " " "
2  ( 1 )  " "  " "      "*"   " "       " "          " "         " "        " "      "*"       " "       " "   " "    " " " "
3  ( 1 )  " "  " "      "*"   "*"       " "          " "         " "        " "      "*"       " "       " "   " "    " " " "
4  ( 1 )  "*"  " "      "*"   "*"       " "          " "         " "        " "      "*"       " "       " "   " "    " " " "
5  ( 1 )  "*"  " "      "*"   "*"       " "          " "         " "        " "      "*"       " "       " "   "*"    " " " "
6  ( 1 )  "*"  " "      "*"   "*"       " "          " "         " "        " "      "*"       " "       "*"   "*"    " " " "
7  ( 1 )  "*"  " "      "*"   "*"       " "          " "         " "        " "      "*"       " "       "*"   "*"    "*" " "
8  ( 1 )  "*"  "*"      "*"   "*"       " "          " "         " "        " "      " "       " "       "*"   "*"    "*" "*"
9  ( 1 )  "*"  "*"      "*"   "*"       " "          " "         " "        " "      "*"       " "       "*"   "*"    "*" "*"
10  ( 1 ) "*"  "*"      "*"   "*"       " "          "*"         "*"        " "      " "       " "       "*"   "*"    "*" "*"
11  ( 1 ) "*"  "*"      "*"   "*"       " "          "*"         "*"        " "      "*"       " "       "*"   "*"    "*" "*"
12  ( 1 ) "*"  "*"      "*"   "*"       "*"          "*"         "*"        " "      "*"       " "       "*"   "*"    "*" "*"
13  ( 1 ) "*"  "*"      "*"   "*"       "*"          "*"         "*"        " "      "*"       "*"       "*"   "*"    "*" "*"
14  ( 1 ) "*"  "*"      "*"   "*"       "*"          "*"         "*"        "*"      "*"       "*"       "*"   "*"    "*" "*"
```

2.2.3. Buidling the final multivariable model (including prior knowledge):

```
> p_ShockWHOo_mid <- log(2)
```

```
> p_ShockWHOo_hi <- log(2.2)

> finalmodel <- stan_glm(RespdistressWHOo ~ ShockWHOo + Coinf + FluidsIn1, data = MicroRDFILE,
family = binomial("logit"))

  prior = normal(location = c(0, p_ShockWHOo_mid, p_ShockWHOo_hi, 0, 0, 0), scale = NULL)

> posteriors <- describe_posterior(finalmodel)

> print_md(posteriors, digits = 2)

> ps1 <- prior_summary(finalmodel)

> ps1$prior

> check_model(finalmodel)

SAMPLING FOR MODEL 'bernoulli' NOW (CHAIN 1).

Chain 1:

Chain 1: Gradient evaluation took 2e-05 seconds

Chain 1: 1000 transitions using 10 leapfrog steps per transition would take 0.2 seconds.

Chain 1: Adjust your expectations accordingly!

Chain 1:

Chain 1:

Chain 1: Iteration:    1 / 2000 [  0%]  (Warmup)

Chain 1: Iteration:  200 / 2000 [ 10%]  (Warmup)

Chain 1: Iteration:  400 / 2000 [ 20%]  (Warmup)

Chain 1: Iteration:  600 / 2000 [ 30%]  (Warmup)

Chain 1: Iteration:  800 / 2000 [ 40%]  (Warmup)

Chain 1: Iteration: 1000 / 2000 [ 50%]  (Warmup)

Chain 1: Iteration: 1001 / 2000 [ 50%]  (Sampling)

Chain 1: Iteration: 1200 / 2000 [ 60%]  (Sampling)

Chain 1: Iteration: 1400 / 2000 [ 70%]  (Sampling)

Chain 1: Iteration: 1600 / 2000 [ 80%]  (Sampling)

Chain 1: Iteration: 1800 / 2000 [ 90%]  (Sampling)

Chain 1: Iteration: 2000 / 2000 [100%]  (Sampling)

Chain 1:

Chain 1:  Elapsed Time: 0.062421 seconds (Warm-up)

Chain 1:                0.067594 seconds (Sampling)

Chain 1:                0.130015 seconds (Total)

Chain 1:


SAMPLING FOR MODEL 'bernoulli' NOW (CHAIN 2).

Chain 2:

Chain 2: Gradient evaluation took 2.3e-05 seconds

Chain 2: 1000 transitions using 10 leapfrog steps per transition would take 0.23 seconds.

Chain 2: Adjust your expectations accordingly!
```

```
Chain 2:
Chain 2:
Chain 2: Iteration:    1 / 2000 [  0%]  (Warmup)
Chain 2: Iteration:  200 / 2000 [ 10%]  (Warmup)
Chain 2: Iteration:  400 / 2000 [ 20%]  (Warmup)
Chain 2: Iteration:  600 / 2000 [ 30%]  (Warmup)
Chain 2: Iteration:  800 / 2000 [ 40%]  (Warmup)
Chain 2: Iteration: 1000 / 2000 [ 50%]  (Warmup)
Chain 2: Iteration: 1001 / 2000 [ 50%]  (Sampling)
Chain 2: Iteration: 1200 / 2000 [ 60%]  (Sampling)
Chain 2: Iteration: 1400 / 2000 [ 70%]  (Sampling)
Chain 2: Iteration: 1600 / 2000 [ 80%]  (Sampling)
Chain 2: Iteration: 1800 / 2000 [ 90%]  (Sampling)
Chain 2: Iteration: 2000 / 2000 [100%]  (Sampling)
Chain 2:
Chain 2:  Elapsed Time: 0.067215 seconds (Warm-up)
Chain 2:                0.064146 seconds (Sampling)
Chain 2:                0.131361 seconds (Total)
Chain 2:


SAMPLING FOR MODEL 'bernoulli' NOW (CHAIN 3).
Chain 3:
Chain 3: Gradient evaluation took 2.7e-05 seconds
Chain 3: 1000 transitions using 10 leapfrog steps per transition would take 0.27 seconds.
Chain 3: Adjust your expectations accordingly!
Chain 3:
Chain 3:
Chain 3: Iteration:    1 / 2000 [  0%]  (Warmup)
Chain 3: Iteration:  200 / 2000 [ 10%]  (Warmup)
Chain 3: Iteration:  400 / 2000 [ 20%]  (Warmup)
Chain 3: Iteration:  600 / 2000 [ 30%]  (Warmup)
Chain 3: Iteration:  800 / 2000 [ 40%]  (Warmup)
Chain 3: Iteration: 1000 / 2000 [ 50%]  (Warmup)
Chain 3: Iteration: 1001 / 2000 [ 50%]  (Sampling)
Chain 3: Iteration: 1200 / 2000 [ 60%]  (Sampling)
Chain 3: Iteration: 1400 / 2000 [ 70%]  (Sampling)
Chain 3: Iteration: 1600 / 2000 [ 80%]  (Sampling)
Chain 3: Iteration: 1800 / 2000 [ 90%]  (Sampling)
Chain 3: Iteration: 2000 / 2000 [100%]  (Sampling)
```

```
Chain 3:
Chain 3:  Elapsed Time: 0.060377 seconds (Warm-up)
Chain 3:                0.05794 seconds (Sampling)
Chain 3:                0.118317 seconds (Total)
Chain 3:


SAMPLING FOR MODEL 'bernoulli' NOW (CHAIN 4).
Chain 4:
Chain 4: Gradient evaluation took 1.3e-05 seconds
Chain 4: 1000 transitions using 10 leapfrog steps per transition would take 0.13 seconds.
Chain 4: Adjust your expectations accordingly!
Chain 4:
Chain 4:
Chain 4: Iteration:    1 / 2000 [  0%]  (Warmup)
Chain 4: Iteration:  200 / 2000 [ 10%]  (Warmup)
Chain 4: Iteration:  400 / 2000 [ 20%]  (Warmup)
Chain 4: Iteration:  600 / 2000 [ 30%]  (Warmup)
Chain 4: Iteration:  800 / 2000 [ 40%]  (Warmup)
Chain 4: Iteration: 1000 / 2000 [ 50%]  (Warmup)
Chain 4: Iteration: 1001 / 2000 [ 50%]  (Sampling)
Chain 4: Iteration: 1200 / 2000 [ 60%]  (Sampling)
Chain 4: Iteration: 1400 / 2000 [ 70%]  (Sampling)
Chain 4: Iteration: 1600 / 2000 [ 80%]  (Sampling)
Chain 4: Iteration: 1800 / 2000 [ 90%]  (Sampling)
Chain 4: Iteration: 2000 / 2000 [100%]  (Sampling)
Chain 4:
Chain 4:  Elapsed Time: 0.066315 seconds (Warm-up)
Chain 4:                0.067286 seconds (Sampling)
Chain 4:                0.133601 seconds (Total)
Chain 4:
```

Table: Summary of Posterior Distribution

Parameter	Median	95% CI	pd	ROPE	% in ROPE	Rhat	ESS
(Intercept)	-5.48	[-8.87, -3.15]	100%	[-0.10, 0.10]	0%	1.000	1710.00
ShockWHOo	2.44	[0.42, 4.73]	99.10%	[-0.10, 0.10]	0%	1.000	2168.00
Coinf	2.01	[0.15, 4.14]	98.35%	[-0.10, 0.10]	0%	1.000	2814.00
FluidsIn1	0.77	[0.11, 1.63]	98.72%	[-0.10, 0.10]	0%	1.000	2355.00

```
$dist
[1] "normal"


$location
[1] 0 0 0


$scale
[1] 2.5 2.5 2.5


$adjusted_scale
[1] 6.315830 5.489890 1.924982


$df
NULL
```

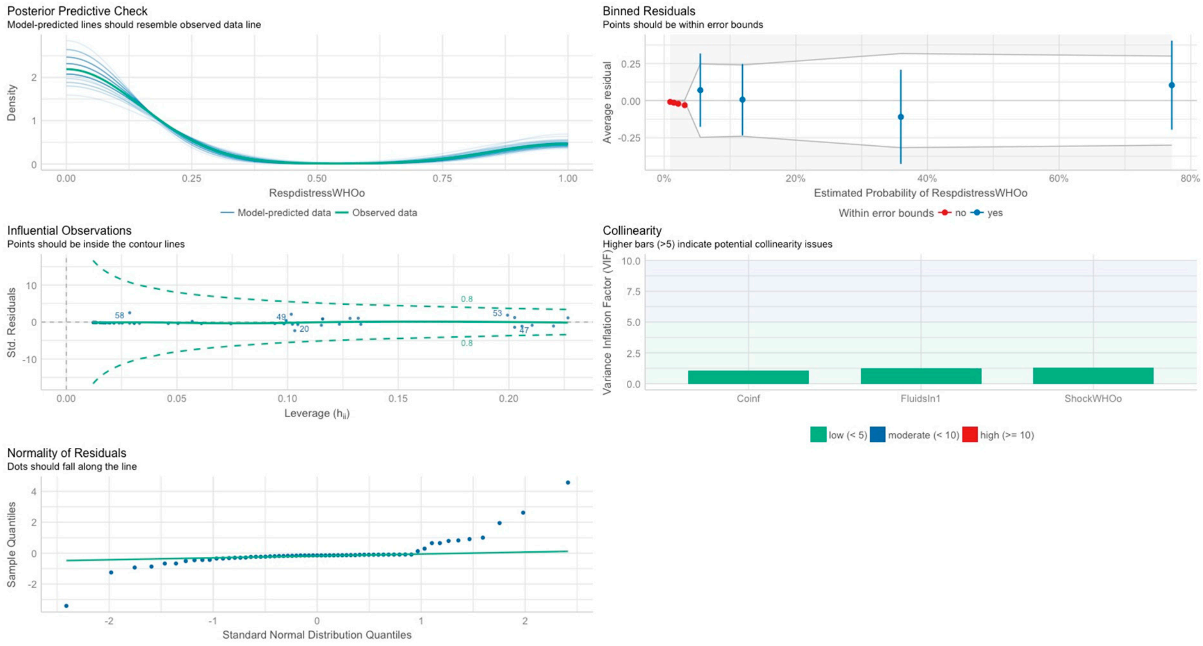

Figure S4. Model diagnostics for the final multivariate Bayesian logistic regression.

2.2.4. Sensitivity analysis – Firth logistic regression of the final model

```
finalmodelf<-logistf(data=MicroRDFILE, RespdistressWHOo ~ ShockWHOo + Coinf + FluidsIn1, 
firth=TRUE, pl=TRUE)

> summary(finalmodelf)

logistf(formula = RespdistressWHOo ~ ShockWHOo + Coinf + FluidsIn1, 

    data = MicroRDFILE, pl = TRUE, firth = TRUE)
```

```
Model fitted by Penalized ML

Coefficients:

                  coef  se(coef)  lower 0.95 upper 0.95     Chisq            p method

(Intercept) -4.6387136 1.1918229 -7.66991804  -2.577070 30.233929 3.829514e-08      2

ShockWHOo    2.0518538 0.9145220  0.23840410   4.115482  4.938308 2.626774e-02      2

Coinf        1.7338358 0.8598094  0.03652136   3.620045  4.007464 4.529924e-02      2

FluidsIn1    0.6333311 0.3299553  0.01098882   1.419793  3.993355 4.568002e-02      2


Method: 1-Wald, 2-Profile penalized log-likelihood, 3-None


Likelihood ratio test=24.58329 on 3 df, p=1.88696e-05, n=63

Wald test = 20.42701 on 3 df, p = 0.0001384385> exp(cbind(OR=coef(model12), confint(model12)))

                     OR    Lower 95%   Upper 95%

(Intercept) 0.009670129 0.0004666561  0.07599638

ShockWHOo   7.782314390 1.2692219791 61.28175069

Coinf       5.662331659 1.0371964594 37.33924383

FluidsIn1   1.883875547 1.0110494150  4.13626282
```

Appendix A – Creating a correlation plot

```
> install.packages("corrplot")

> library(corrplot)

corrplot 0.92 loaded

> head(APORFILE_01_23)

> res <- cor(APORFILE_01_23)

> round(res, 2)

Warnmeldung:

In cor(APORFILE_01_23) : Standardabweichung ist Null
```

	JaundiceWHOo	HyperparasitemiaWHOo	RenalimpWHOo	ShockWHOo	RespdistressWHOo	BleedingWHOo	AnemiaWHOo	AcidosisWHOo	ComaWHOo	HypoglyWHOo	ConvulWHOo
JaundiceWHOo	1.00	0.18	0.36	0.28	0.14	0.17	0.17	0.30	0.30	0.18	NA
HyperparasitemiaWHOo	0.18	1.00	-0.02	0.16	0.10	0.03	0.03	0.02	0.13	-0.07	NA
RenalimpWHOo	0.36	-0.02	1.00	0.49	0.53	0.24	0.35	0.73	0.48	0.26	NA
ShockWHOo	0.28	0.16	0.49	1.00	0.53	0.46	0.35	0.35	0.60	0.26	NA
RespdistressWHOo	0.14	0.10	0.53	0.53	1.00	0.16	0.38	0.51	0.51	0.28	NA
BleedingWHOo	0.17	0.03	0.24	0.46	0.16	1.00	0.18	0.27	0.41	0.29	NA
AnemiaWHOo	0.17	0.03	0.35	0.35	0.38	0.18	1.00	0.54	0.41	0.29	NA
AcidosisWHOo	0.30	0.02	0.73	0.35	0.51	0.27	0.54	1.00	0.36	0.36	NA
ComaWHOo	0.30	0.13	0.48	0.60	0.51	0.41	0.41	0.36	1.00	0.36	NA
HypoglyWHOo	0.18	-0.07	0.26	0.26	0.28	0.29	0.29	0.36	0.36	1.00	

```
> M = cor(APORFILE_01_23)

  corrplot(M, method = 'number')
```

or

```
> M = cor(APORFILE_01_23)
  corrplot(M, method = 'circle')
```

Appendix B - Creating boxplots

```
> install.packages("gplots")
> library(gplots)
> boxplot2(FluidsInR~RespdistressWHOo, data=RDFILE, main="Remaining time on ICU", frame = FALSE, top = TRUE, xlab="Complication", ylab="Fluid volume (mL/kg/h)",ylim = c(0, 8), col=c("white", "light gray", "dark gray"), border="black"))
```

Supplementary File S2. STROBE Statement checklist

	Item No.	Recommendation	Page No.	Relevant text from manuscript
**Title and abstract**	1	(*a*) Indicate the study's design with a commonly used term in the title or the abstract	1	Abstract: "This retrospective observational investigation…"
		(*b*) Provide in the abstract an informative and balanced summary of what was done and what was found	1	
**Introduction**				
Background/rationale	2	Explain the scientific background and rationale for the investigation being reported	1-2	Currently, shortages of medication, material, and, most importantly, nurses, interfere with medical care in many European institutions as a result of the coronavirus pandemic. Such shortages may negatively influence patient outcomes such as mortality, readmissions, and length of hospital and ICU stay (ICU-LOS).
Objectives	3	State specific objectives, including any prespecified hypotheses	2	The study aimed to identify complications associated with prolonged ICU-LOS among patients with falciparum malaria imported to Berlin, Germany, in the pre-pandemic era and to determine targets for their prevention.
**Methods**				
Study design	4	Present key elements of study design early in the paper	2-3	Retrospective analysis of all cases eligible during the study period. The study is a secondary analysis of a previous observational study.
Setting	5	Describe the setting, locations, and relevant dates, including periods of recruitment, exposure, follow-up, and data collection	2-3	Location: Charité University Hospital, Berlin, a tertiary-care teaching hospital. Study period: January, 1st 2001 through December, 31st 2015.
Participants	6	(*a*) *Cohort study*—Give the eligibility criteria, and the sources and methods of selection of participants. Describe methods of follow-up<br>*Case-control study*—Give the eligibility criteria, and the sources and methods of case ascertainment and control selection. Give the rationale for the choice of cases and controls<br>*Cross-sectional study*—Give the eligibility criteria, and the sources and methods of selection of participants	2-3	All eligible cases ≥18 years of age hospitalized with slide-proven imported falciparum malaria were enrolled (Flow chart: Figure 1, page 3). No follow-up was required according to the study design.
		(*b*) *Cohort study*—For matched studies, give matching criteria and number of exposed and unexposed<br>*Case-control study*—For matched studies, give matching criteria and the number of controls per case		
Variables	7	Clearly define all outcomes, exposures, predictors, potential confounders, and effect modifiers. Give diagnostic criteria, if applicable	4	a) Primary outcome of interest: ICU length of stay (ICU-LOS), calculated in hours.

				b) Secondary outcome of interest: risk factors for individual malaria-specific complications associated with prolonged ICU-LOS. The WHO 2022 definition for severe falciparum malaria with minor modifications was applied.
Data sources/ measurement	8*	For each variable of interest, give sources of data and details of methods of assessment (measurement). Describe comparability of assessment methods if there is more than one group	2-3	For all patients standardized electronic files with detailed information on sociodemographics, travel history, full medical history including prior malaria episodes, current medication, results of physical examination and laboratory investigations were available. Data capture was therefore high.
Bias	9	Describe any efforts to address potential sources of bias		
Study size	10	Explain how the study size was arrived at	2	All eligible cases ≥18 years of age hospitalized with slide-proven imported falciparum malaria during the study period were enrolled. When patients were treated more than once during the study period repeated episodes were excluded from the analysis.

Quantitative variables	11	Explain how quantitative variables were handled in the analyses. If applicable, describe which groupings were chosen and why	4-5	To identify malaria-specific complications associated with prolonged ICU-LOS conventional Cox proportional hazard regression with censoring of cases discharged after the median of ICU-LOS (i.e., by 61 hours) was used in univariate analysis as well as multivariate analysis. To ensure the robustness of the results, sensitivity analyses with discharge after 38 and 91 hours (i.e., after the first and the third quartile of ICU-LOS) as endpoints were also performed.<br>For variable selection the best subset selection method was used. Important potential confounding factors were included in the multivariate model. The results of the final multivariate model were reported as adjusted hazard ratios (aHRs). The proportional hazard assumption was tested for each covariate in the final

				multivariate model and for the global model the Schoenfeld residual test was used. Influential observations were tested by dfbeta values. The statistical significance level was set at 5% for all analyses.<br><br>For the identification of risk factors for individual malaria-specific complications Bayesian logistic regression was used in order to minimize sparse data bias. A frequentist approach (Firth's logistic regression) was also performed as sensitivity analysis for each covariate and for the final multivariate model. The best combination of predictor variables for the final multivariate model was again selected employing the best subset selection method. Prior information was included in the analysis where available. If no prior information was available default (weakly informative) priors were used. Significance of the parameters was tested by the Region of Practical Equivalence (ROPE) test. Model diagnostics included tests for influential observations, normality of residuals, and collinearity. The median of the posterior distribution with its 95% credibility intervals was reported for parameters with significant associations in multivariate analysis. The effect existence was described by the probability of direction (pd).
Statistical methods	12	(*a*) Describe all statistical methods, including those used to control for confounding	3-4	See above (item #11).
		(*b*) Describe any methods used to examine subgroups and interactions		Not applicable.
		(*c*) Explain how missing data were addressed	10-11	There were no missing data for the outcome of interest "ICU-LOS".<br>For the secondary outcome of interest "risk factors for respiratory distress" some covariates had missing values as described in the footnote of table 5.
		(*d*) *Cohort study*—If applicable, explain how loss to follow-up was addressed<br>*Case-control study*—If applicable, explain how matching of cases and controls was addressed<br>*Cross-sectional study*—If applicable, describe analytical methods taking account of sampling strategy		Not applicable.

		(*e*) Describe any sensitivity analyses	3-4, 10-11	For both study endpoints sensitivity analyses were performed in order to ensure the robustness of the results. For ICU-LOS sensitivity analyses with discharge after 38 and 91 hours (i.e., after the first and the third quartile of ICU-LOS) as endpoints were performed. Only malaria-specific complications with significant association in all three intervals were included in the final multivariate model. For identification of risk factors for individual malaria-specific complications Firth's logistic regression was performed for each covariate and for the final multivariate model as sensitivity analysis.
**Results**				
Participants	13*	(a) Report numbers of individuals at each stage of study—eg numbers potentially eligible, examined for eligibility, confirmed eligible, included in the study, completing follow-up, and analysed	3	Reported under section 3.1. as well as summarized in Figure 1.
		(b) Give reasons for non-participation at each stage	3	In case that a patient was treated more than once with falciparum malaria in the institution during the study period only the first episode was included in the analysis. Accordingly, 22 of such repeat episodes of falciparum malaria were excluded.
		(c) Consider use of a flow diagram	3	A flow diagram is presented in Figure 1.
Descriptive data	14*	(a) Give characteristics of study participants (eg demographic, clinical, social) and information on exposures and potential confounders	10-11	Summarized in Table 5.
		(b) Indicate number of participants with missing data for each variable of interest	10-11	For each covariate absolute numbers and percentages of the total study population or the median with the inter-quartile range are given in Table 5. All missing values are listed in the footnote of table 5.
		(c) *Cohort study*—Summarise follow-up time (eg, average and total amount)		Not applicable.
Outcome data	15*	*Cohort study*—Report numbers of outcome events or summary measures over time	5, 6, 7, 10-11	Reported under sections 3.1. and 3.2. as well as in figure 2, figure 3, table 2, table 3, and table 5.
		Case-control study—Report numbers in each exposure category, or summary measures of exposure		Not applicable.
		Cross-sectional study—Report numbers of outcome events or summary measures		Not applicable.
Main results	16	(*a*) Give unadjusted estimates and, if applicable, confounder-adjusted estimates and their precision (eg, 95% confidence interval). Make clear which confounders were adjusted for and why they were included	7-11	a) Primary outcome of interest "ICU-LOS": section 3.2., unadjusted hazard ratios with 95%CIs are given in table 3, adjusted hazard ratios with 95%CIs are given in table 4.<br><br>b) Secondary outcome of interest "risk factors for respiratory distress": section

				3.3., table 5 (unadjusted) and table 6 (adjusted hazard ratios with 95% credibility intervals).
		(*b*) Report category boundaries when continuous variables were categorized		Not applicable.
		(*c*) If relevant, consider translating estimates of relative risk into absolute risk for a meaningful time period		Not applicable.

Other analyses	17	Report other analyses done—eg analyses of subgroups and interactions, and sensitivity analyses	3-4, 10-11	Sensitivity analyses were an important part of the statistical investigation. Their results are summarized in tables 3 and 5 (please refer to section 12e).
**Discussion**				
Key results	18	Summarise key results with reference to study objectives	7, 8, 9, 11	Key results of the analysis are:<br><br>(1) Respiratory distress was the only malaria-specific complication independently associated with prolonged ICU-LOS (section 3.2. and table 4).<br><br>(2) Shock, co-infections, and higher fluid volumes administered on day 1 of admission were associated with development of respiratory distress (section 3.3. and table 6).
Limitations	19	Discuss limitations of the study, taking into account sources of potential bias or imprecision. Discuss both direction and magnitude of any potential bias	14	The main limitations of the study are its retrospective, single-centre design, the long observation period, and the relatively small case numbers predisposing to sparse data bias. This limits the quality of the data and thus generalizability.
Interpretation	20	Give a cautious overall interpretation of results considering objectives, limitations, multiplicity of analyses, results from similar studies, and other relevant evidence	11-14	Refer to the "Discussion" section.
Generalisability	21	Discuss the generalisability (external validity) of the study results	14	Generalisability is limited as discussed in the "limitations" section.
**Other information**				
Funding	22	Give the source of funding and the role of the funders for the present study and, if applicable, for the original study on which the present article is based		This work received no funding.

*Give information separately for cases and controls in case-control studies and, if applicable, for exposed and unexposed groups in cohort and cross-sectional studies.

Note: An Explanation and Elaboration article discusses each checklist item and gives methodological background and published examples of transparent reporting. The STROBE checklist is best used in conjunction with this article (freely available on the Web sites of PLoS Medicine at http://www.plosmedicine.org/ (accessed on 30 April 2020), Annals of Internal Medicine at http://www.annals.org/ (accessed on 30 April 2020), and Epidemiology at http://www.epidem.com/, (accessed on 30 April 2020). Information on the STROBE Initiative is available at www.strobe-statement.org.
